# Seasonal variation in gut microbiota composition: cross-sectional evidence from Ukrainian population

**DOI:** 10.1186/s12866-020-01786-8

**Published:** 2020-04-21

**Authors:** Alexander Koliada, Vladyslav Moseiko, Mariana Romanenko, Liubov Piven, Oleh Lushchak, Nadiia Kryzhanovska, Vitaly Guryanov, Alexander Vaiserman

**Affiliations:** 1 Institute of Gerontology, Vyshgorodskaya st. 67, Kyiv, 04114 Ukraine; 2 Molecular Genetic Laboratory Diagen, Kyiv, Ukraine; 3 Vasyl Stefanyk Precarpathian National University, Ivano-Frankivsk, Ukraine; 4 PB MEDICOM-IN, Dnipro, Ukraine; 5 Bogomolets National Medical University, Kyiv, Ukraine

**Keywords:** Gut microbiota composition, Seasonality of sampling, Diet, Lifestyle factors

## Abstract

**Background:**

Gut microbiota composition is known to depend on environmental (diet, day length, infections, xenobiotic exposure) and lifestyle (alcohol/drug intake, physical activity) factors. All these factors fluctuate seasonally, especially in areas with highly variable climatic conditions between seasons. Seasonal microbiota changes were reported in several previous studies. The purpose of our study was to investigate whether there is a seasonal variability in the gut microbiota composition in Ukrainian population. In contrast to previous studies performed on small-size samples using a longitudinal design, we used cross-sectional design with a large sample size (*n* = 769). Determination of microbial composition at the level of major microbial phyla was performed by qRT-PCR.

**Results:**

The relative abundance of major taxonomic groups of gut microbiota was found to be affected by month of sampling. Actinobacteria were more abundant and Bacteroidetes were less abundant in summer-derived samples compared to those obtained during other seasons, whereas Firmicutes content was seasonally independent. The Firmicutes to Bacteroidetes (F/B) ratio was significantly higher in summer-derived samples than in winter-derived ones. Odds to have F/B > 1 were 3.3 times higher in summer samples and 1.9 times higher in autumn samples than in winter ones; neither age, nor sex were significant confounding factors.

**Conclusions:**

Seasonality of sampling could influence results of human microbiome research, thereby potentially biasing estimates. This factor must be taken into consideration in further microbiome research.

## Background

The term “gut microbiota” generally refers to a dynamic community of about 100 trillion microbial cells harbored within the human gastrointestinal tract, and the term “human microbiome” refers to the about 3 million genes (mostly from bacteria) harbored by these microbial cells [1–3]. The most common human intestinal bacteria are members of the gram-negative Bacteroidetes and the gram-positive Firmicutes phyla, and also some other phyla, such as Actinobacteria, Fusobacteria and Verrucomicrobia, that are present at subdominant levels [4]. It is becoming increasingly clear now that most microbes inhabiting our body are critically involved in the organism’s vital functions. The microbiota contains far more important metabolic genes than have been discovered in the human genome and provides the host organism with essential enzymes and biochemical pathways. The metabolic processes regulated by intestinal microbial communities are substantially contributed to both nutrient acquisition and xenobiotic processing, including the metabolism of undigested carbohydrates and vitamin biosynthesis [5, 6]. Moreover, the microbiota provides a barrier protecting the host against infections through production of antimicrobial compounds and competitive exclusion of pathogens [7]. The unbalanced state of the gut microbiota composition (dysbiosis) can result in adverse health outcomes including not only intestinal diseases but also extra-intestinal pathological conditions such as atherosclerosis, metabolic syndrome, diabetes mellitus and cancer [8–10].

The composition of microbiota is known to depend on both host genetic background and lifestyle/environmental factors, although recent evidence suggest that exogenous determinants dominate over host genetics in shaping the human gut microbiota [11]. Dietary factors certainly play a central role in these processes [12, 13], although environmental (day length, infections, xenobiotic exposure) and lifestyle (alcohol/drug intake, physical activity) factors are undoubtedly of great importance as well [14, 15]. All these variables obviously fluctuate with season, especially in areas with highly variable climatic conditions between seasons. Given this, it is not surprising that seasonal changes in the intestinal microbiota composition have been reported in several studies. Most of these findings were obtained from isolated religious groups or autochthonous hunter-gatherer communities. Such variations were found, for example, in the study by Davenport et al. conducted in a population of Hutterites, members of an ethno-religious anabaptist group having similar lifestyles due to their communal life [16]. In particular, since they live and eat together, their dietary patterns are practically the same across individuals and very stable throughout the year, with the exception of availability of fresh fruits and vegetables throughout the summer and autumn months. Although overall gut microbiome stability was observed within individuals over time, the significant and consistent population-wide shifts in their gut microbiome composition have been evident across seasons. More specifically, significantly increased ratios of Bacteroidetes and decreased ratios of Actinobacteria and Firmicutes were found in summer compared to winter fecal samples. Subsequently, seasonal variations in the gut microbiota composition were observed in this population by using a genome-wide association study (GWAS) approach [17]. At least eight bacterial taxa have been identified whose seasonal abundance was associated with single nucleotide polymorphisms in the host genome. Seasonal changes in the gut microbiome, in which some taxa become undetectable only to reappear in a subsequent season, were also observed in the Hadza hunter-gatherers of Tanzania [18]. More recently, the seasonal shifts in bacterial taxa, diversity, and also in carbohydrate utilization by the microbiota have been found in the same hunter-gatherer group [19]. No seasonal changes in the microbiome composition were, however, found in another hunter-gatherer society, such as the Inuit from the Canadian Arctic, perhaps due to an increasingly westernized diet [20]. Nevertheless, despite the lack of directional seasonal shifts, Inuit microbiomes have been shown to be more variable over time in both diversity and composition, than those of their urbanized counterparts from Montréal. Evidence for seasonal variation in intestinal microbiota was also found in Japanese and Mongolian populations [21, 22]. Remarkably, in the Mongolian study, seasonal differences in microbiota composition were more pronounced in the Khentii pasturing area where dietary pattern is simple and varied during the year than in TUW province and Ulan Bator where dietary structure is diverse and stable throughout the year [22]. In addition, evidence for population-level seasonal microbiome shifts came from several indirect observations such as seasonal modulation of body odors, which are known to be associated with microbiota composition, in the U.S. population [23].

The purpose of our study was to investigate whether there is a seasonal variability in the gut microbiota composition in Ukrainian population. In contrast to previous studies on the topic performed on small-size samples using a longitudinal design, we used cross-sectional design with a large population-based sample in our study.

## Results

**Fig. 1:**
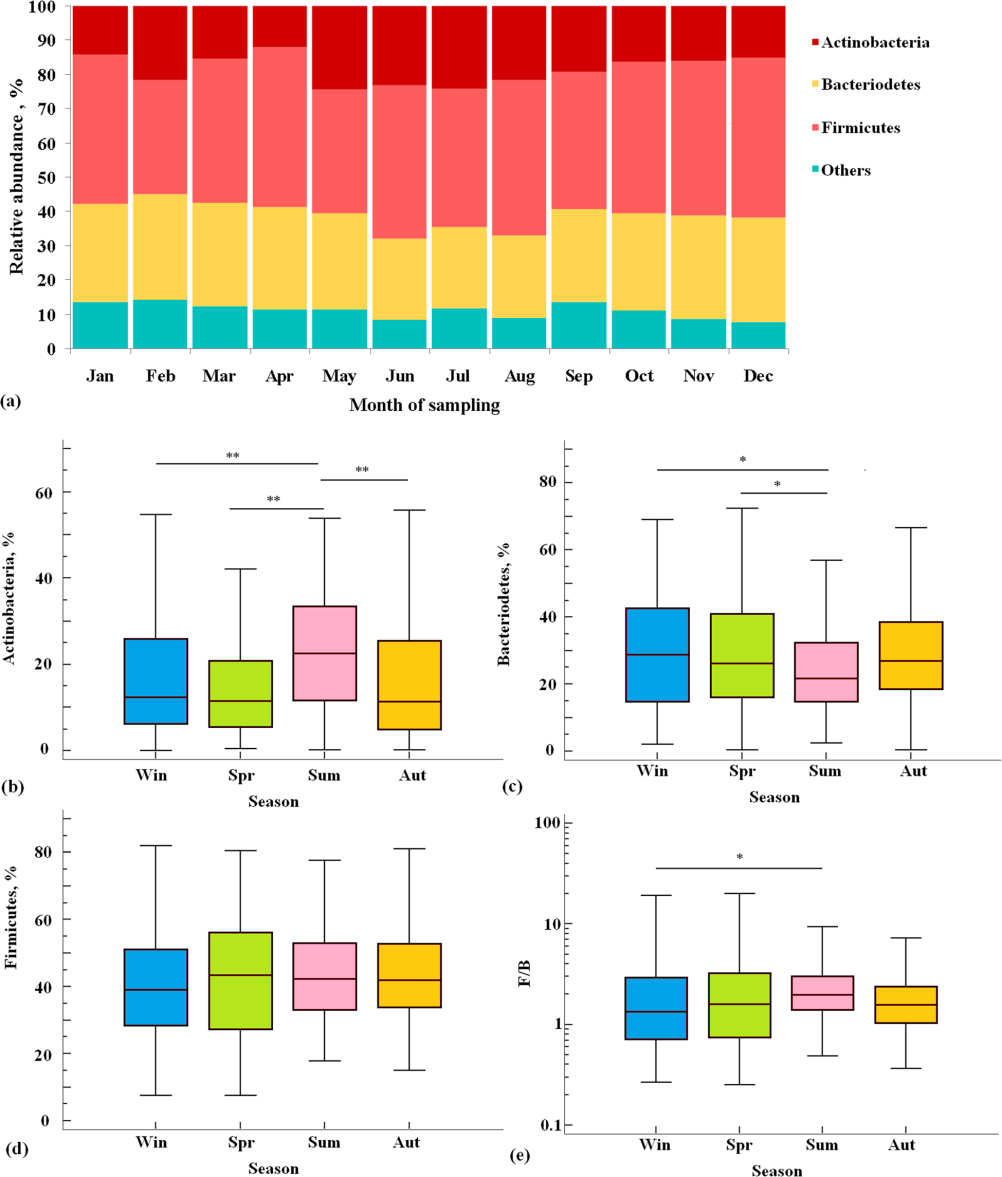
Relative abundance of major gut microbiota phyla in Ukraine population, by month of sampling (**a**). Relative abundance of Actinobacteria (**b**), Bacteroidetes (**c**), Firmicutes (**d**), and F/B ratio (**e**) in Ukrainian population, by season of stool sampling. In each box-and-whisker plot, the box represents the values from the lower to upper quartile (25 to 75 percentile). The middle line inside the box represents the median. The whiskers above and below the box show the locations of the minimum and maximum values. * *p* < 0.05, ** *p* < 0.01, according to the post-hoc Dunn’s test

The relative abundance of major taxonomic groups of the gut microbiota at the phylum level was found to be substantially affected by month of sampling (Fig. 1a). In particular, relative abundance of Actinobacteria was found to be significantly dependent on season of sampling (Kruskal-Wallis test, *p* < 0.001). Actinobacteria was much more abundant in summer samples compared to samples derived in any other season (Fig. 1b). The Bacteroidetes content was also found to be seasonally dependent (Kruskal-Wallis test, *p* = 0.014). Contrary to Actinobacteria, it was significantly reduced in the summer-derived samples compared to the winter- and spring-derived samples (Fig. 1c). The Firmicutes content was seasonally independent in our study (Fig. 1d; Kruskal-Wallis test, *p* = 0.31). Accordingly, the Firmicutes to Bacteroidetes (F/B) ratio which is widely used as a biomarker for pathological conditions [24], was shown to be dependent on season of sampling (Kruskal-Wallis test, *p* = 0.025), with this ratio being significantly higher in summer-derived samples than in winter-derived ones (Fig. 1e).

**Fig. 2:**
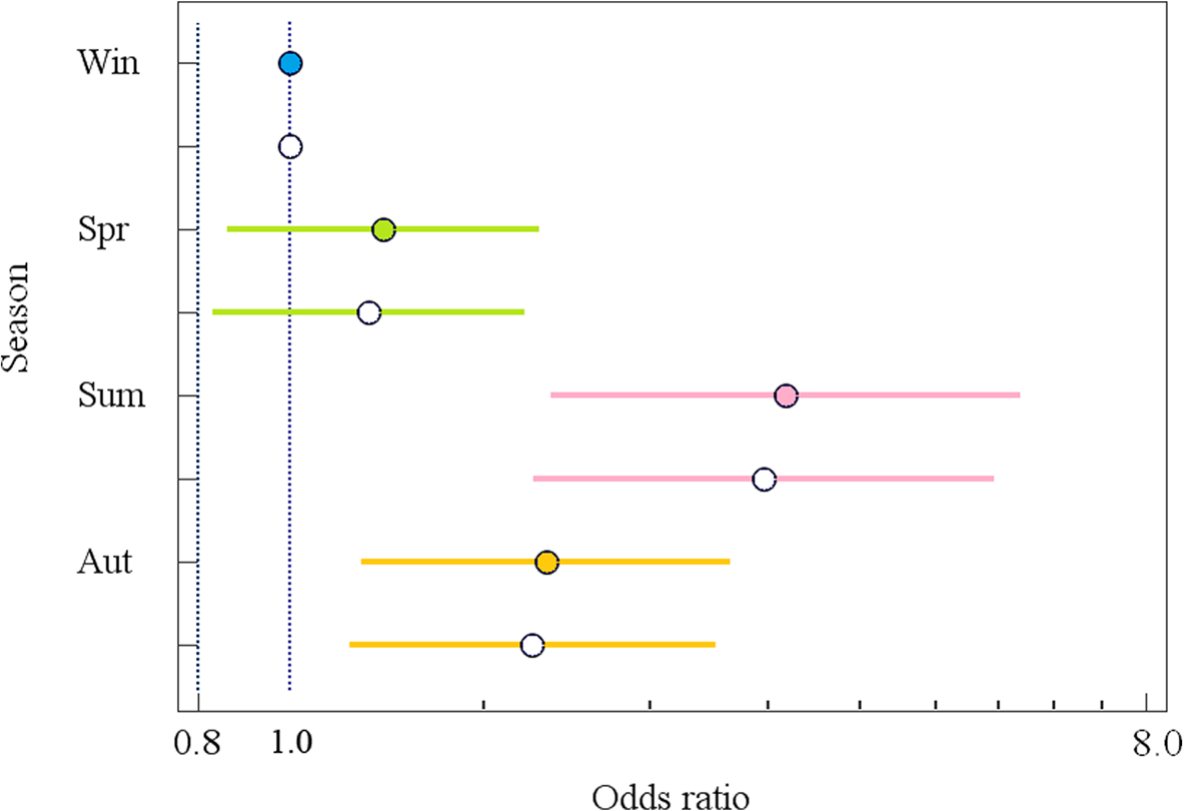
Odds ratios and 95% confidence intervals to have F/B > 1 in fecal samples obtained in different seasons. F/B ratio in the winter-derived samples refers to the reference level (OR = 1). Unadjusted and adjusted for age and sex values are indicated by filled and open circles, respectively

The logistic regression model was also used to estimate the effect of season of sampling on the F/B ratio. As seen from Table 1, in an unadjusted model, the odds to have F/B > 1 was 3.3 times higher in summer samples and 1.9 times higher in autumn samples than in winter ones (see also Fig. 2 for illustration). In the age- and sex-adjusted model, these odds ratios (ORs) were almost unchanged: OR = 3.2 for summer samples and OR = 1.8 for autumn samples. The results obtained indicate that neither age, nor sex were significant confounding factors.

**Table 1:** Logistic regression models of the effect of season of sampling on the F/B ratio

Variables	Regression coefficient, b ± m	OR (95% CI)	***p***	AUC (95% CI)
**One-factor model, Season**				
Season				
Winter	Reference			0.60 (0.56–0.64)
Spring	0.23 ± 0.19	1.26 (0.86–1.84)	0.24	
Summer	1.20 ± 0.29	3.34 (1.89–5.90)	< 0.001	
Autumn	0.62 ± 0.23	1.86 (1.19–2.91)	0.006	
**Three-factor model, Season + Sex + Age**				
Season				
Winter	Reference			0.62 (0.58–0.65)
Spring	0.19 ± 0.20	1.21 (0.82–1.77)	0.34	
Summer	1.15 ± 0.29	3.17 (1.78–5.62)	< 0.001	
Autumn	0.59 ± 0.23	1.80 (1.15–2.82)	0.01	
Sex				
Female	Reference			
Male	−0.19 ± 0.17	0.82 (0.59–1.15)	0.26	
Age				
0–19	Reference			
20–39	0.43 ± 0.25	1.54 (0.94–2.52)	0.08	
40–59	0.41 ± 0.25	1.50 (0.91–2.47)	0.11	
≥60	0.490 ± 0.36	1.63 (0.80–3.30)	0.17

In Table 1: Event, F/B > 1, No event, F/B < 1.

**Fig. 3:**
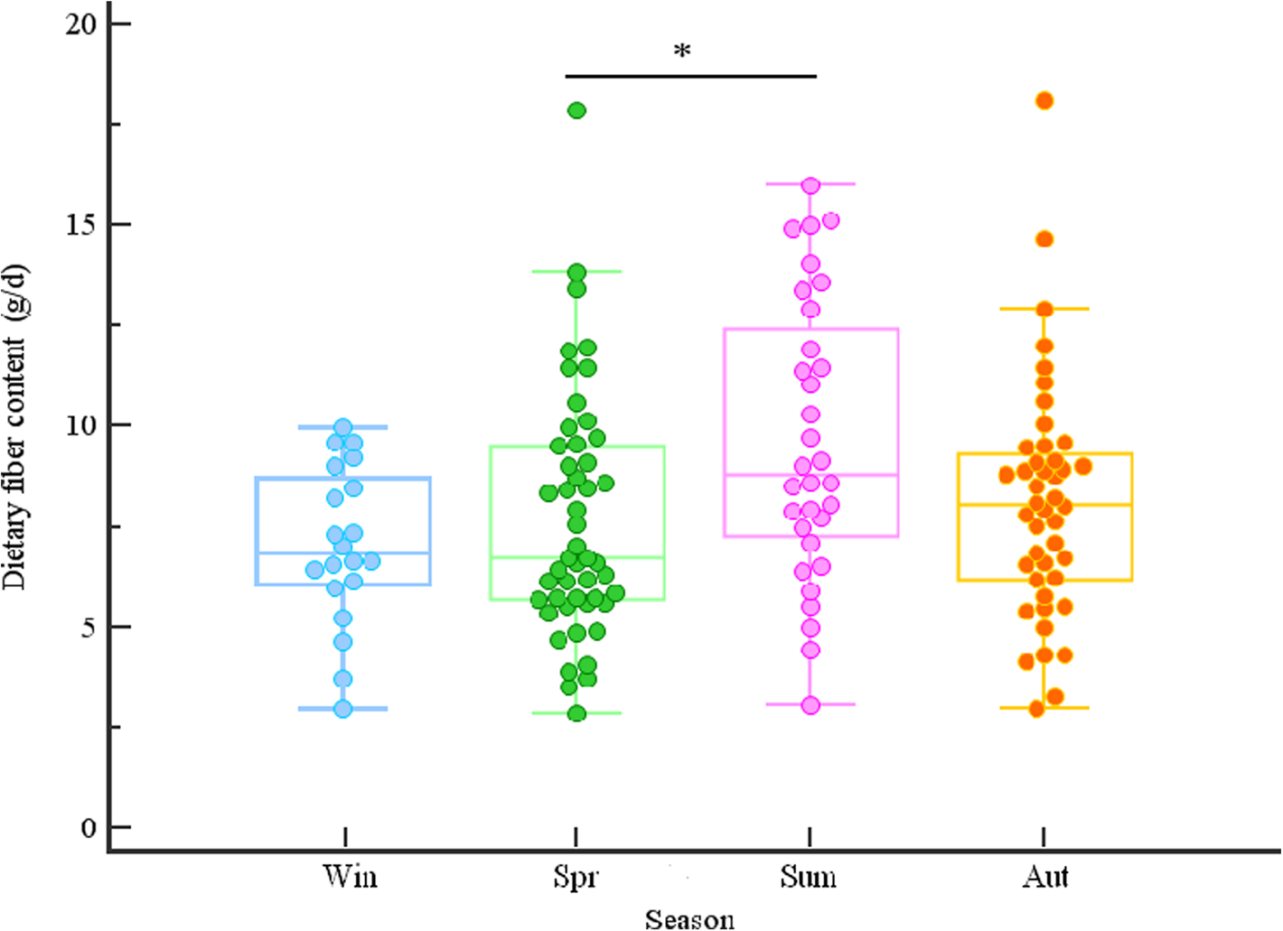
Dietary fiber content (g/d), according to the season of stool sampling. Dots represent individual data points. Lines extend from the minimum to the maximum values, excluding “outside” values (that are larger than the upper quartile plus 1.5 times the interquartile range); these values are displayed as separate points. Other figure details are the same as in Fig. 1

Since the shifts in the gut microbial community composition might be largely attributable to the changes in nutrient availability, the dietary patterns were assessed in 145 study participants by dietary questionnaires. Significant differences among seasons were observed in dietary fiber intake (Kruskal-Wallis test, *p* = 0.03). Higher levels of fiber consumption were found in the summer (see Fig. 3 for illustration) while no seasonal differences were observed for other polysaccharide components such as hemicellulose and pectin, as well as for all polysaccharides in total (*p* > 0.05 in all cases).

## Discussion

In our study, seasonal shifts in the gut microbiota composition were observed in Ukrainian population. This was, however, not unexpected as this population, being currently low-income and largely agricultural, still essentially depends on climatic factors. As a result, it differs significantly from most modern developed societies that depend only a little on seasonal factors, in particular, due to the year-round availability of fresh fruits and vegetables from a global market. Consequently, different food categories may be consumed homogeneously across the whole calendar year. The latter, however, can lead to adverse health outcomes as well, since the season-independent dietary patterns could be related to a loss of microbiome diversity required for maintaining normal metabolism and immune function. In addition, the Western-style diet associated with reduced fiber intake and excess intake of sucrose and saturated fats can result in an insufficient supply of dietary prebiotics necessary to promote the growth of important bacterial taxa [25, 26]. Season-dependent dietary patterns may, on the contrary, promote gut microbiota diversity. For example, in the Davenport et al. study [16], the summer diet, rich in fresh fruits and vegetables and being therefore high-fiber and high-carbohydrate, has been associated with enhanced levels of Bacteroidetes, while the levels of Actinobacteria have been found to be decreased in Hutterite gut microbiomes. Seasonal changes in gut microbiome composition among the Tanzanian Hadza were also shown to be related to the dietary seasonal variation [18]. Throughout the dry season, the Hadza diet is restricted to meat and (primarily) tubers rich in dietary fiber, whereas during the wet season, the Hadza predominantly eat baobab fruits, berries, and honey, which leads to a decreased fiber content in their diet. The seasonal changes observed in the Hazdas’ gut are likely aimed at digestive conditions. Throughout the wet season, when most calories come from the honey and fiber intake is substantially reduced, the diversity of the Hazdas’ microbiome drops sharply, and their microbiomes become largely enriched with microbial enzymes specifically designed to utilize the fructan, a sugar contained in berries.

In our research, similarly to the study by Davenport and co-authors [16], the summer diet was high-fiber, according to dietary records. Such dietary pattern, however, has not been associated with enhanced levels of Bacteroidetes in the gut microbiome. These levels, on the contrary, were found to be reduced in the summer samples. The inconsistency of our data with results obtained by Davenport et al. [16] can likely be explained by the fact that much more complicated and ambiguous relationships than a simple correlation between a particular food component and certain microbial phylum (e.g., dietary fiber content and Bacteroidetes abundance) exist between seasonal variables and microbiota composition. Indeed, recent evidence indicates that modifications of human gut microbiota by certain non-digestible dietary carbohydrates occur at the particular strain and species levels and cannot be easily predicted a priori [27, 28]. In addition, not only dietary, but also non-dietary factors such as outdoor temperature, day length, sunlight exposure, infection exposures (winter-time respiratory infections vs summer-time gastrointestinal infections) etc. may likely influence the gut microbiome composition. In previous studies, gut microbiome has been indeed shown to be substantially modulated by seasonally fluctuating factors such as vitamin D supply from cutaneous synthesis and nutritional intake [29], bacterial and helminth infections [30], antibiotic exposures [31], as well as the physical activity level [32]. In addition, along with differences in seasonal patterns in the Ukrainian population and in the Hutterite population, the contradiction between our findings and those reported by Davenport et al. [16] could also be potentially explained by the difference in the methods used, namely, real-time polymerase chain reaction (qRT-PCR) vs 16S rRNA gene sequencing. This, however, requires further study.

The findings from our study and other evidence for seasonality of gut microbiota seem to be important because the seasonal shifts in microbiota composition can potentially contribute to the seasonal pattern of incidence and recurrence, firmly established not only for infectious but also for some non-infectious diseases like affective disorders [33], schizophrenia [34], stroke [35], and also ischemic heart disease and heart failure [36]. Seasonal factors operating perinatally can also influence health status and disease risk in adulthood (so-called “seasonal programming” phenomenon) [37]. This phenomenon was repeatedly observed across countries. For example, in Ukraine, epidemiological findings indicate that the risk for developing diabetes mellitus (both type 1 and type 2) may be substantially influenced by seasonal factors operating in the perinatal period [38, 39]. The seasonally-driven factors could be potentially important contributors to the process of microbial gut colonization in early life and, by influencing symbiotic host-bacterial interactions, affect the disease risks in adult life [40, 41].

The strength of our research lies in the fact that, unlike previous studies conducted with small sample sizes, we used a large sample size. The important limitation of this study is that, owing to the cross-sectional design used, only limited inferences about causal relationships between risk factors and obtained outcomes may be drawn. Therefore, we plan to conduct a further study in Ukrainian population with a longitudinal design which allows more powerful inferences about causal relationships among the study variables.

## Conclusions

In conclusion, our results indicate that composition of the human intestinal microbiota can vary seasonally. Thereby, the results of human microbiome research might be potentially influenced by seasonality of sampling. The neglect of this factor could lead, in some cases, to biased estimates of observed associations. Therefore, this factor must be taken into consideration in further microbiome research.

## Methods

### Study population

The fecal samples were obtained throughout the period from 19 May, 2017 to 13 May, 2019 from 769 subjects residing in Ukraine and visited medical clinics in Kyiv (mainly, residents of North-Western regions of Ukraine) and Dnipro (mainly, residents of South-Eastern regions of Ukraine) for consultation and laboratory examination. Written informed consents to provide a stool sample and to the availability of the stored samples for additional bioassays were obtained from all the study participants before enrollment. The inclusion criterion was permanent residence in Ukraine for at least 2 years before visiting. The exclusion criteria were as follows: (a) refusal to give informed consent; (b) serious health problems such as recent surgery, current presence of cancer or infectious disease, acute relapse of any chronic disease, mental illness, types 1 diabetes or poorly controlled type 2 diabetes, and also current use of prohibited medications, probiotics, antibiotics and immunosuppressants. All recruited persons were asked to maintain their usual lifestyle, including dietary and physical activity habits, throughout the study period. The regular communication between investigators and study participants ensured good compliance. After the study finished, recommendations were given to all participants to correct their lifestyle and dietary habits. The study was carried out in accordance with the Declaration of Helsinki and approved by Ethics Committee of the Kyiv Institute of Gerontology (approval number: 88/16; date of approval: 28/12/2016).

Basic demographic characteristics of recruited subjects are presented in Table 2.

**Table 2:** Baseline characteristics of the study participants

Age group	Female, n (%)	Male, n (%)	All, n (%)
0–19	52 (10.3)	44 (16.6)	96 (12.4)
20–39	205 (40.7)	113 (42.6)	318 (41.4)
40–59	186 (36.9)	97 (36.6)	283 (36.8)
60+	61 (12.1)	11 (4.2)	72 (9.4)
**Total**	504 (65.5)	265 (34.5)	769

### Dietary recording

Completed 7-day weighed dietary records were provided by 145 study participants. For the calculation of nutrient composition, dietary records (515 food products in total) were analyzed with RATIONAL NUTRITION TEST TRP-D02 software (version 2008; scientific-productive company «VIRIA», Ukraine).

### Sample collection and DNA extraction

Fresh morning-collected stool samples were provided once by each subject in a stool container on site.

Fecal samples were collected and frozen immediately upon defecation and all collected aliquots were stored at − 20 °C for 1 week until DNA isolation. DNA was extracted from 1.5–2 frozen stool aliquots using the phenol-chloroform method by protocol [42]. DNA was finally eluted in 200 μl elution buffer. The DNA quantity and quality was measured by NanoDrop ND-8000 (Thermo Scientific, USA). Samples with a DNA concentration less than 20 ng or an A 260/280 less than 1.8 were subjected to ethanol precipitation to concentrate or further purified, respectively, to meet the quality standards.

### PCR amplification

PCR reaction was performed in real-time thermal cycler Rotor-Gene 6000 (QIAGEN, Germany) as described previously with modification [43]. The PCR reaction conditions consisted of an initial denaturing step of 5 min at 95 °C, 30 cycles of 95 °C for 15 s, annealing (15 s at 61.5 °C and 72 °C at 30 s), and a final elongation step at 72 °C for 5 min. Every PCR reaction contained 0.05 units/μl of Taq polymerase (Sigma Aldrich), 0.2 mM of each dNTP, 0.4 μM of each primer, 1× buffer, ~ 10 ng of DNA and water to 25 μl. Samples were amplified with all primer pairs in triplicates. The Cts (univ and spec) were the threshold cycles registered by the thermocycler. The average Ct value obtained for each pair was transformed into percentage by the formula given by Bacchetti De Gregoris et al. [44]:

$$ \mathrm{X}={\left(\mathrm{Eff}.\mathrm{Univ}\right)}^{\mathrm{Ctuniv}}/{\left(\mathrm{Eff}.\mathrm{Spec}\right)}^{\mathrm{Ctspec}}\ast 100 $$

where Cts (univ and spec) are the threshold cycles registered by the thermocycler, Eff. Univ is the calculated effectiveness of universal primers (2 = 100% and 1 = 0%) and Eff. Spec refers to the effectiveness of taxon-specific primers. According to this equation, X represents the percentage of 16S taxon-specific copy number presenting in a given sample.

### Identification of microbial composition

Determination of microbial composition at the level of major microbial phyla was performed by a method described by Bacchetti De Gregoris et al. [44] with quantitative real-time PCR (qRT-PCR), using universal primers targeting bacterial 16S rRNA gene, and also primers specific for Firmicutes*,* Actinobacteria and Bacteroidetes. The primer sequences used are presented in Table 3.

**Table 3:** Primer nucleotide sequences used in qRT-PCR assay

Phylum	Primer nucleotide sequence	
	Forward	Reverse
Bacteroidetes	798cfbF AAACTCAAAKGAATTGACGG	cfb967R GGTAAGGTTCCTCGCGCTAT
Firmicutes	928F-firm TGAAACTYAAGGAATTGACG	1040FirmR ACCATGCACCACCTGTC
Actinobacteria	Act920F3 TACGGCCGCAAGGCTA	Act1200R TCRTCCCCACCTTCCTCCG
*16S rRNA gene*	926F AAACTCAAAKGAATTGACGG	1062R CTCACRRCACGAGCTGAC

### Statistical analysis

Statistical analyses were performed using the software STATISTICA 11.0. Shapiro–Wilk test was performed to test the normality of the distribution of all the quantitative variables studied. Since variables did not follow normal distribution, non-parametric methods were selected for further analysis of the data. To identify the statistical difference among different seasonal groups, median abundances of each phylum were compared by the Kruskal-Wallis test. Pairwise multiple comparisons between groups were performed using post-hoc Dunn’s test. A multivariate logistic regression analysis was used to evaluate the effect of season of sampling on the Firmicutes to Bacteroidetes (F/B) ratio. The receiver operating characteristic (ROC) curve analysis was used to evaluate the logistic regression models. In this analysis, the power of the values predicted by the model to discriminate between negative and positive cases was quantified by the area under the ROC curve (AUC) [45].

## Data Availability

The datasets generated and analyzed during the study are available upon reasonable request to the corresponding author.

## Abbreviations

F/B ratio: Firmicutes to Bacteroidetes ratio
GWAS: genome-wide association study
OR: odds ratio
qRT-PCR: real-time polymerase chain reaction
